# A Case of Treatment With a Combination of Covered Stents and Artificial Blood Vessel for Iliac Artery Rupture During Transcatheter Aortic Valve Replacement

**DOI:** 10.1002/ccr3.70523

**Published:** 2025-05-20

**Authors:** Wensheng Zhu, Hanqing Deng, Weiqing Hu, Shixun Wang

**Affiliations:** ^1^ School of Clinical Medicine Shandong Second Medical University Weifang China; ^2^ Department of Cardiology Weifang People's Hospital (The First Affiliated Hospital of Shandong Second Medical University) Weifang Shandong China; ^3^ Department of Vascular Surgery Weifang People's Hospital (The First Affiliated Hospital of Shandong Second Medical University) Weifang Shandong China

**Keywords:** aortic stenosis, artery reconstruction, iliac artery rupture, transcatheter aortic valve replacement, vascular complication

## Abstract

Vascular complications (VCs) associated with Transcatheter Aortic Valve Replacement (TAVR), particularly iliac artery rupture, pose significant challenges. We report a case of complete iliac artery disruption during TAVR. A combination of a covered stent and artificial blood vessel repair was employed to achieve revascularization of the iliac artery. In this case, the patient was placed on bed rest for 10 days to avoid stent dislodgement due to early movement and was discharged from the hospital 13 days later. After that, the patient stayed away from significant lower limb activity for 3 months after surgery and underwent enhanced follow‐up. Regular follow‐up indicated that the patient recovered well, with no significant discomfort in the right lower limb and consistent pulse in the dorsal foot artery on both sides. The treatment of such complications typically involves surgical intervention, while this method shows a new viable option for managing such VCs. By using this method, blood flow can be blocked more quickly than through surgery, which reduced the risk of massive bleeding, death, and limb loss and improved the prognosis.

**Summary**

- This case demonstrates the effective management of iliac artery rupture during TAVR using a combination of covered stents and an artificial blood vessel, highlighting a viable approach for vascular complications.

## 1 Introduction

Aortic stenosis (AS) is a prevalent valvular heart disease among the aging population. The prognosis for patients with severe AS combined with heart failure is poor, and medical treatment does not improve the prognosis [1]. Transcatheter Aortic Valve Replacement (TAVR) and Surgical Aortic Valve Replacement (SAVR) are cutting‐edge treatments for aortic stenosis [2]. Although the risk of serious vascular complications (VCs) is higher with TAVR compared to SAVR, TAVR has been demonstrated to be non‐inferior to SAVR in reducing clinical events (including death and disabling stroke) in high‐ or intermediate‐risk surgical patients [3, 4, 5].

Initially, apical access was the only alternative for TAVR. However, with advancements in TAVR, additional access routes such as trans‐iliofemoral, trans‐aortic, trans‐carotid, trans‐caval, trans‐axillary, and trans‐septal are now available [6]. The trans‐femoral approach is the first choice in all guidelines and consensus due to its wide application and low rate of vascular complications [7, 8, 9].

In this case, the patient successfully received the VitaFlow valve prosthetic system (MicroPort, Shanghai, China) using a 22F femoral artery sheath. Although the valve functioned well, vascular damage occurred during the sheath removal process, leading to massive blood loss and hemorrhagic shock. The patient was stabilized through fluid replacement, norepinephrine administration, blood transfusion, and vascular reconstruction using an artificial blood vessel and covered stent. The patient ultimately improved and was discharged from the hospital 13 days later. This technique represents a viable option for managing iliac artery avulsion following TAVR and is likely to improve outcomes after that issue.

## 2 Case History

**FIGURE 1:**
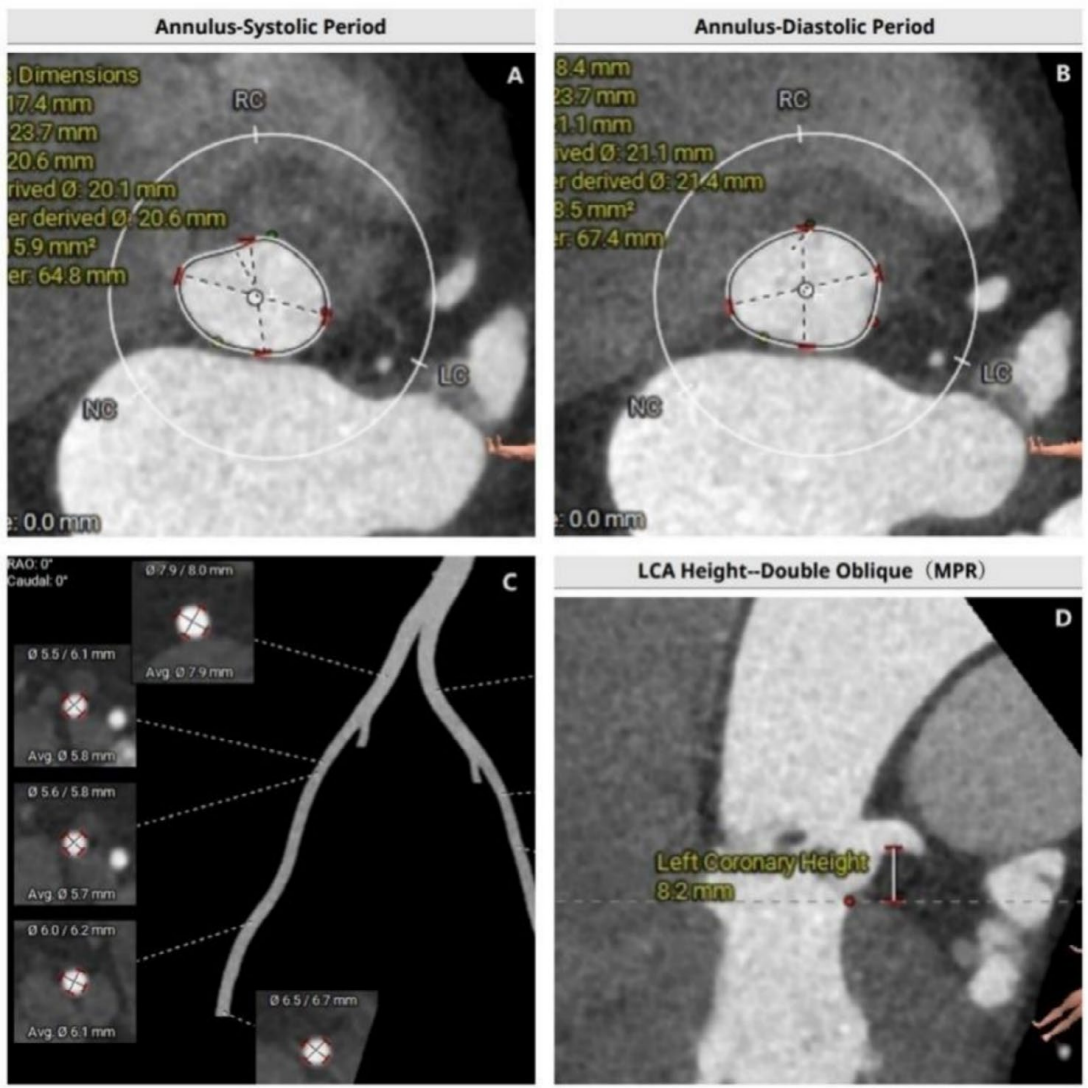
The assessment by computed tomography angiography (CTA). (A) Annulus‐Systolic Period (20.6 mm). (B) Annulus‐Diastolic Period (21.4 mm). (C) The minimum (narrowest) lumen diameter (5.7 mm). (D) Left Coronary Height (8.2 mm).

A 69‐year‐old woman with a history of hypertension and coronary artery disease (CAD) was diagnosed with severe aortic stenosis (AS) and heart failure (HF) due to frequent chest pain. Echocardiography revealed that the aortic valve area was 0.51 cm^2^, the peak velocity was 4.7 m/s, the maximum transvalvular pressure was 89 mmHg, and the mean gradient was 50 mmHg. The patient refused Surgical Aortic Valve Replacement (SAVR) and opted for Transcatheter Aortic Valve Replacement (TAVR). Based on the preoperative assessment of access arteries and coronary CTA (Figure 1), the femoral artery was confirmed as the access route for the transfemoral approach.

## 3 Methods

The patient was brought to the interventional operating room, prepped, and draped. Following routine intravenous anesthetic induction, orotracheal intubation was performed. A pacemaker electrode was implanted in the right ventricle and tested successfully.

**FIGURE 2:**
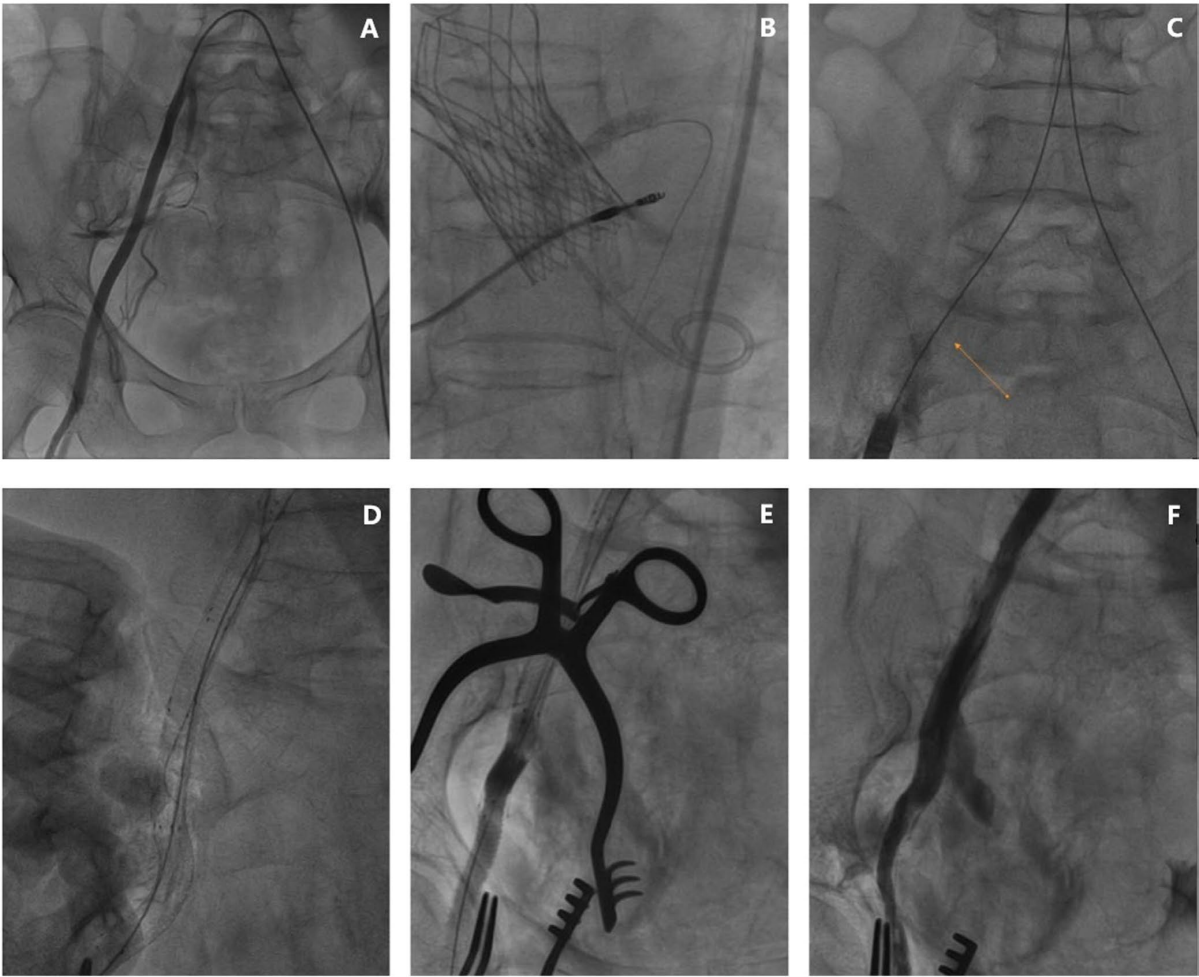
Intraoperative digital subtraction angiography (DSA). The surgical procedure is as follows: (A) Shows the right femoral artery approach by the left femoral artery angiography. (B) Shows well positioned transcatheter aortic valve prosthesis. (C) As withdrew the sheath, arrows refer to the leakage of the contrast. (D) Angiography shows stent‐grafts (covered stent) positioned and working well. (E) Endograft bridging covered stent and artificial vessel. (F) Angiography shows the grafts patency and no leakage of the contrast medium.

The right femoral artery was punctured under the guidance of left femoral artery angiography (Figure 2A). After heparin anticoagulation, a 20F femoral introducer sheath was inserted into the right femoral artery, but it was not possible to advance the 21 mm VitaFlow valve prosthetic system (MicroPort, Shanghai, China) to the iliac artery. The sheath was replaced with a 22F femoral sheath, and the valve was successfully advanced to the standard position and deployed. The left coronary artery chimney stenting technique was used to reduce the risk of coronary occlusion. Aortic root angiography and ultrasonography confirmed that the valves were well positioned and functioning properly, with mild regurgitation and a transvalvular pressure near zero (Figure 2B).

**FIGURE 3:**
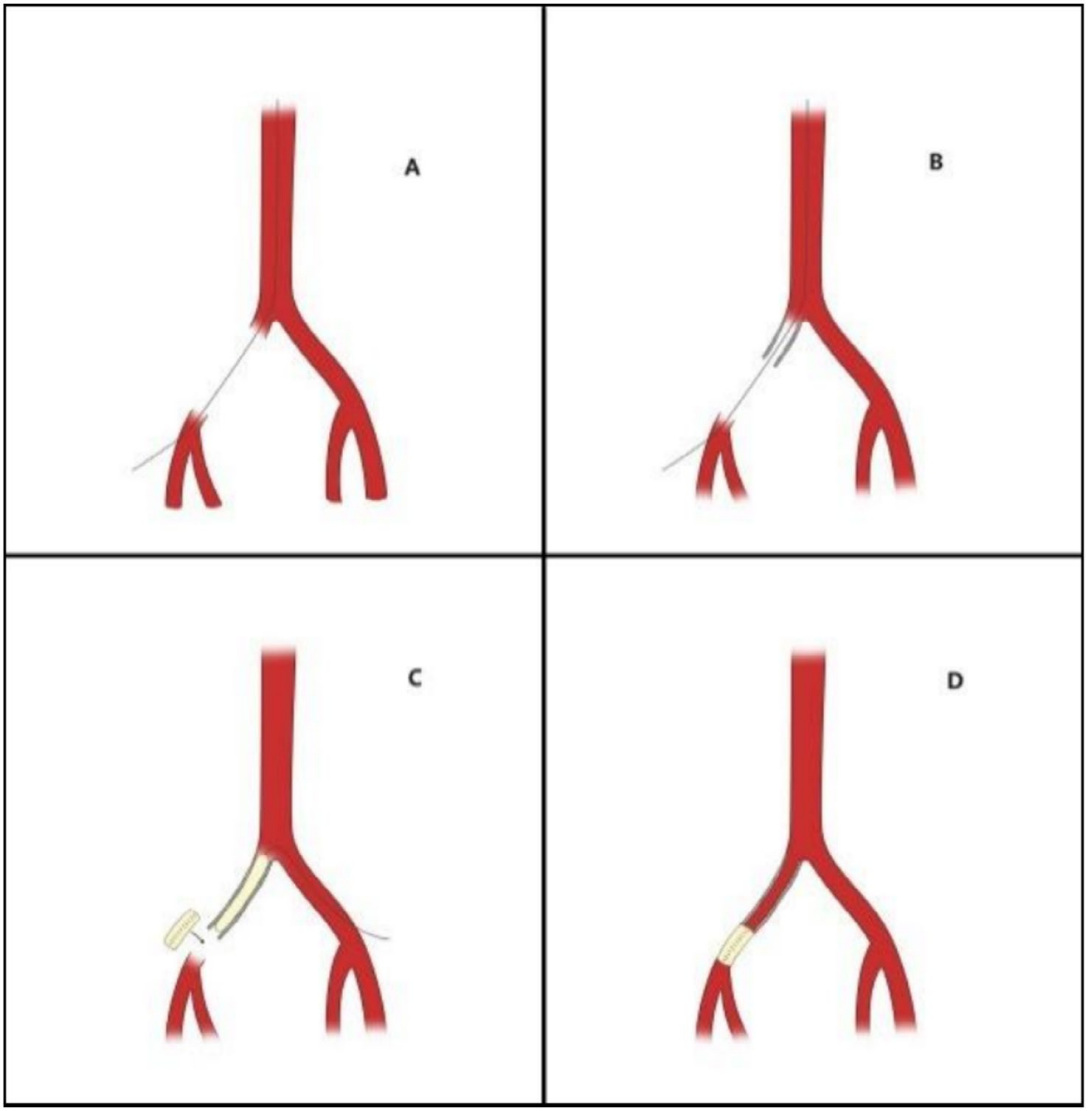
Schematic of surgery. (A) The right iliac artery is completely disrupted intraoperatively. (B) Covered stent implanted. (C) Balloon expansion to block blood flow and a graft was sutured to the distal end of the covered stent. (D) The graft was then sutured to the stump of the superficial femoral artery; vascular reconstruction completed.

During the removal of the sheath, it became stuck and could not be withdrawn. Nitroglycerin and a calcium channel blocker (diltiazem) were administered intravenously to address arterial vasospasm, but these measures did not achieve the desired effect. The sheath was withdrawn to the level of the common iliac artery; it suddenly slipped, resulting in a rupture of the iliac artery (Figures 2C and 3A). A rapid drop in blood pressure ensued, prompting immediate fluid resuscitation and norepinephrine infusion. Concurrently, external compression was applied to the right abdomen to control bleeding.

An 8.0 mm × 50 mm VIABAHN covered stent (W.L. Gore and Associates, Flagstaff, AZ, USA) and another 8.0 mm × 100 mm VIABAHN covered stent were placed in the right common iliac artery and the external iliac artery, respectively, but they failed to connect. A 7.0 mm × 100 mm VIABAHN covered stent was then placed between the first two stents to establish a connection (Figure 3B), which was successfully achieved (Figure 2D). An 8.0 mm × 100 mm Mustang balloon (Boston Scientific, Natick, MA, United States) was inserted through the left sheath and expanded within the covered stent to block blood flow (Figure 3C). A 10‐cm longitudinal skin incision was made at the right inguinal ligament to expose the distal end of the superficial femoral artery, where an atraumatic vascular clamp was used to stop the bleeding. The proximal end of the stent was freed, and a 6 mm artificial blood vessel was sutured to the distal end of the covered stent (Figures 2E and 3C).

Following the removal of the Mustang balloon, angiography revealed thrombus formation in the area of the covered stent. A 4F Fogarty embolectomy catheter (Edwards Lifesciences, Irvine, CA) was used to remove the thrombus from the reconstructed iliac artery and superficial femoral artery. After thrombus removal, the blood flow in the distal superficial femoral artery returned to normal.

The graft was then sutured to the stump of the superficial femoral artery, completing the reconstruction of the right iliofemoral artery. Re‐angiography showed that the lumen of the right iliofemoral artery was reconstructed satisfactorily, with normal shape and blood flow velocity, and no contrast leakage was observed (Figures 2F and 3D).

Due to the patient's B Rh‐negative blood type and preoperative analysis indicating a low risk of bleeding, no routine blood preparation was made. After intraoperative treatment of the vascular complications, the patient was transferred to the intensive care unit (ICU), fluid resuscitation and blood transfusion were continued. The patient was extubated and transferred to the general ward 1 day after the operation and was advised to remain on bed rest and avoid significant lower limb activity. After receiving postoperative care, the patient's condition improved, and she was discharged 13 days later.

## 4 Conclusion and Results

This case demonstrates the feasibility of managing similar VC in TAVR by inserting a balloon from the lateral approach to block blood flow and then revascularization, including covered stent and artificial blood vessel, but further studies are needed to confirm its safety, efficacy, and prognosis for patients.

## 5 Discussion

Although the incidence of vascular complications during TAVR procedures has gradually decreased with the application of newer generation transcatheter heart valve (THV) systems [10], VCs are a frequent issue during TAVR, with literature reports indicating that the most critical vascular complications are often accompanied by severe bleeding and uncontrolled hemorrhagic shock [10], which should have garnered significant attention from doctors.

In this case, the minimum lumen diameter was 5.7 mm. Although there was no calcification, the potential risk of vascular complications was taken into consideration. Consequently, preoperative preparations included the use of covered stents, a blood vessel‐blocking balloon, and the presence of a vascular surgeon. Additionally, we collaborated with the transfusion department to ensure appropriate blood preparation. Despite the patient's relatively rare blood type, the transfusion team promptly arranged for blood reserves from neighboring cities, ensuring that the patient received the necessary blood supply in a timely manner. In the centers with limited resources, a multidisciplinary team can comprehensively evaluate the patient's condition from different perspectives, leading to more precise treatment plans. Organize multidisciplinary team discussions to optimize surgical plans and minimize the risk of intraoperative complications. Team collaboration enables a rapid response to intraoperative emergencies, ensuring patient safety. By implementing these measures, it is possible to maximize surgical success rates and ensure patient safety even in the face of resource shortages and technological limitations. The above description highlights the crucial role of a multidisciplinary heart valve team and emphasizes the importance of a structured, collaborative approach at TAVR centers. Additionally, preoperative evaluation, individualized decision‐making regarding valve system and vascular approach, and the skills of the TAVR team are essential for successful implantation.

Reports of iliac artery rupture during TAVR are rare. A report from the USA [11] describes a patient who suffered an iliac artery injury; the repair was achieved through percutaneous covered stents implantation. Reports of iliac artery rupture during TAVR are rare. Another case from Japan [12] found that during the advancement of the valve system through the femoral artery, the artery ruptured. However, blood loss was minimized because the valve system blocked blood flow. The ruptured femoral artery was subsequently reconstructed through surgical intervention using an artificial blood vessel graft. In this case, the artery was fully severed, rendering covered stents inadequate due to unlocatable vessel ends and a high risk of occluding the deep or superficial femoral artery. Another case from the USA [13] utilized a similar technique, with similarly favorable clinical results. To manage such complications, an alternative surgical approach involves using a transcatheter aortic block balloon to stop aortic blood flow during repair surgery, but this has certain disadvantages. Firstly, the operating room was not equipped with a conventional aortic balloon, leading to delays in preparing surgical supplies, which increased the patient's risk. Secondly, bleeding from the iliac artery could persist due to blood supply from the aorta's lateral branches. This method is similar to the Crossover Balloon Occlusion Technique (CBOT), described by Sharp et al. [14]; it involves inflating a crossover balloon from the left femoral artery to block blood flow. When managing such vascular complications in centers with limited resources, this case illustrates an effective alternative solution achieved without a transcatheter aortic block balloon and immediate access to vascular surgeons. In summary, implanting covered stents in the proximal segment of the ruptured iliac artery and using CBOT to block blood flow is the most rapid and effective method, providing sufficient time for artificial blood vessel implantation. By reducing the risks of hemorrhagic shock, death, and limb loss, it offers a promising approach to enhancing outcomes in similar cases.

Since the blood vessels were completely severed, it was crucial to enhance stability and minimize the risk of stent dislodgement caused by positional activities. The overlap of covered stents must be substantial, especially at the connection between the first covered stent and the artery's proximal end, which should be long and of a large diameter to ensure a secure fit. The patient should remain on bed rest after surgery to avoid stent dislodgement due to early movement. In this case, the patient was placed on bed rest for 10 days, avoided significant lower limb activity for 3 months post‐surgery, and underwent strengthened follow‐up. Regular follow‐up indicated that the patient recovered well, with no significant discomfort in the right lower limb and consistent pulse in the dorsal foot artery on both sides. This case suggests the feasibility of this method.

## Author Contributions

**Wensheng Zhu:** conceptualization, data curation, formal analysis, writing – original draft. **Hanqing Deng:** formal analysis, writing – review and editing. **Weiqing Hu:** writing – original draft. **Shixun Wang:** conceptualization, funding acquisition, resources, supervision, writing – review and editing.

## Consent

Written informed consent was obtained from the patient to publish this report in compliance with the journal's patient consent policy.

## Conflicts of Interest

The authors declare no conflicts of interest.

## Data Availability

To protect patient privacy, the data is not published publicly. Contact the corresponding author's email address; more clinical data is available on request.
